# A Feasibility Study to Evaluate *Bacillus subtilis* as a Host for Producing Recombinant Human Parathyroid Hormone

**Published:** 2018

**Authors:** Mahdi Karimi, Farida Behzadian, Hamideh Rouhaninejad, Sanaz Yari

**Affiliations:** Department of Molecular Genetics, Research Centre for Biosciences and Biotechnology, Malek-Ashtar University of Technology, Tehran, Iran

**Keywords:** *Bacillus subtilis*, hPTH, Teriparatide

## Abstract

**Background:**

Biosynthetic teriparatide (1–34) (TPD) is a N-terminally truncated version of human parathyroid hormone (hPTH). The recombinant form of this polypeptide has been expressed in *Escherichia coli (E. coli)* and approved as the first anabolic treatment of osteoporosis in the EU and the USA. Feasibility of expression and secretion of a tag- fused form of TPD into *Bacillus subtilis (B. subtilis)* was examined due to several advantages of *B. subtilis* over *E. coli* in production of recombinant proteins with pharmacological activities.

**Methods:**

A codon optimized gene containing TPD open reading frame carrying enterokinase site in its upstream was fully synthesized. According to our cloning scheme, this synthetic polynucleotide was used as a template for PCR amplification using engineered primers in such a way that a polyhistidin tag was added in frame to the upstream of the amplicon as well as two restriction sites at its ends. The resulted amplicon, a cassette containing His-tag, enterokinase site and TPD, from 5′ to 3′, was cloned into pTZ57R/T vector and subjected to sequencing.The cassette was then subcloned into pHT43 shuttle vector and transformed into *B. subtilis*. Expression of target protein was analyzed by SDS-PAGE and western blotting upon induction by IPTG.

**Results:**

The accuracy of construction of pHT43-TPD was confirmed by sequencing and restriction map analyses. SDS-PAGE and western blotting results showed that the recombinant fusion form of hPTH was successfully expressed and secreted into cytoplasm and extracellular medium.

**Conclusion:**

TPD may be successfully expressed and secreted in *B. subtilis*; however, optimization of expression conditions is required for enhancing target protein yield.

## Introduction

Osteoporosis, as a persistent skeletal disorder involves decreased bone strength due to density reduction and increased fracture risk leading to an inequality in normal bone remodeling process where bone mass is resorbed by osteoclasts while failing to create adequate space. As a result, bone microarchitecture becomes porous and susceptible to fracture due to a compromise in its structural integrity 1, 2.

Currently, recommened osteoporosis therapy involves reducing bone remodeling by inhibiting hone resorption. This treatment is not sufficient in decreasing osteoprosis rates of morbidity and mortality 3. In contrast to anti-resorptive medication, anabolic treatment preferentially increases bone formation through direct early stimulation of osteoblasts 4.

Parathyroid Hormone (PTH) and its analogue Teriparatide (TPD) provide the possibility of improving skeletal microarchitecture and represent a new class of anabolic therapies for the treatment of severe osteoporosis 3.

Anabolic properties of full-length PTH are incorporated in TPD as a recombinant human parathyroid hormone (1–34) corresponding to the first 34 amino acids of hPTH 5. Considering costs, side effects, and limited clinical utility, TPD is more effective on patients with severe osteoporosis compared to bisphosphonates and other antiresorptives 6.

Gram-positive bacterium *Bacillus subtilis (B. subtilis)* has been widely adopted for protein production due to its “Generally Recognized As Safe” (GRAS) status in biotechnological processes 7. It demonstrates excellent fermentation properties, strain deficiency in 6 extracellular proteases, complete lack of toxic by-products, and high product yields (20–25 *g/L*) without a significant codon bias 8. Additionally, gram-positive bacterium *B. subtilis* has a naturally high secretory capacity and exports proteins directly into extracellular medium simplifying downstream purification and prevents formation of inclusion bodies 9.

Due to the above-mentioned advantages of *B. subtilis* over *Escherichia coli (E. coli)* in production of recombinant proteins with pharmacological activities, in the present study, a designed tag fused TPD gene was cloned and used to transform *B. subtilis* for the first time. The obtained recombinant *B. subtilis* is capable of expression and particularly secretion of TPD into medium.

## Materials and Methods

### Cassette design

A cassette encoding recombinant human parathyroid hormone favouring high-yield protein production was constructed based on prokaryotic codon usage. This sequence was synthesized by Bioneer Corporation (Bioneer, Korea) and was cloned into pET32a plasmid named pET32a-TPD. From 5′ to 3′, the cassette consisted of enterokinase site to cleave the fusion moiety and the open reading frame encodes for TPD.

### Polymerase chain reaction (PCR)

**Figure 1.**
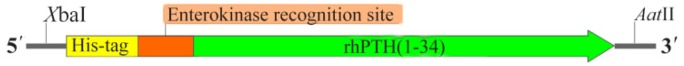
Schematic representation of tag fused hPTH (1–34) (TPD) demonstrating the arrangement and composition of cassette after PCR.

Full-length sequence of TPD was amplified from pET32a-TPD through PCR reaction using Taq polymerase (Thermo, USA). The forward primer with a *XbaI* cut site and a His-tag overhang (5′ATTATCT AGACACCACCACCACCACCATGATGATGATGA TAAGAGCGTGAG3′) and reverse primer with a *Aa-tII* cut site and stop codon overhang (5′GCGCGACG TCTTAGAAGTTATGCACATCCTG3′) were designed in accordance with the cloning strategy and were used to amplify TPD gene from pET32a-TPD vector. Thirty rounds of amplification were performed per following cycle: pre-denaturing step at 94°*C* for 3 *min* proceeded by 30 cycles of denaturation at 94°*C* for 30 *s*, anannealing step at 55°*C* for 30 *s*, and an extension step at 72°*C* for 30 *s*. Final extension step at 72°*C* for 5 *min* was performed. Figure 1 demonstrates schematic drawing of the resulted amplicones with a size of 158 *bp*.

### Cloning and subcloning of TPD amplicon

**Figure 2.**
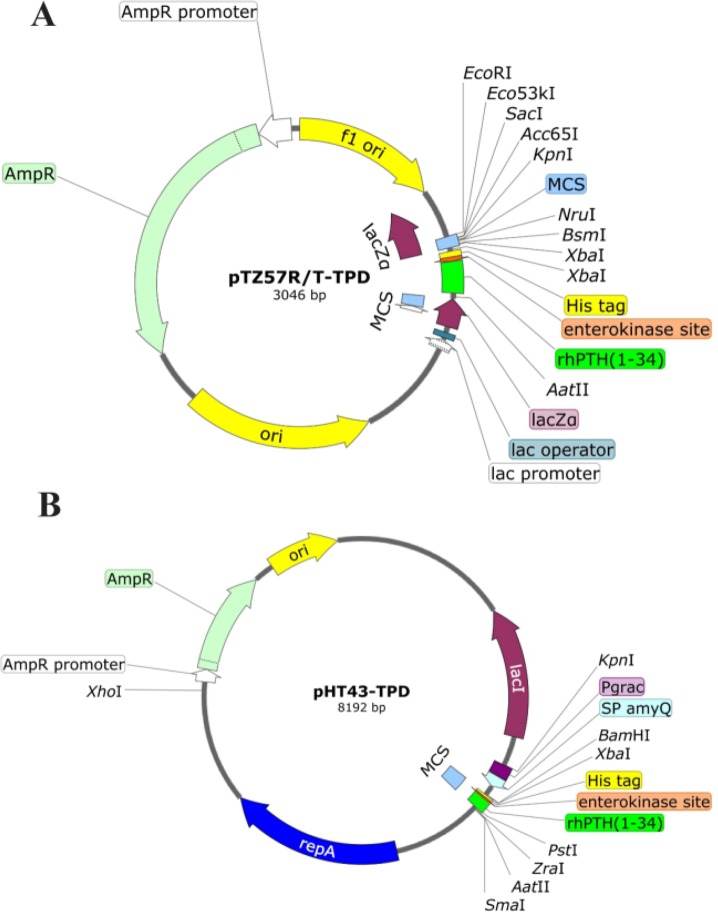
Schematic diagram of recombinant pTZ57R/T-TPD and pHT43-TPD vectors. A) Map of cloned TPD cassette into pTZ57R/T vector. B) The map of subcloned TPD cassette into pHT43. Above diagrams were generated using SnapGene® software from GSL Biotech.

Amplified fragments were purified and cloned into pTZ57R/T vector using the InsTAclone PCR Cloning Kit (Thermo, USA). Purified amplified fragments were then transformed to *E. coli XL1blue* competent cells. Plasmids were purified from insert positive clones and then digested with *XbaI/AatII* after colony PCR screening from white colonies. The released inserts were re-cloned into expression vector pHT43 to construct pHT43-TPD (Figure 2). The fidelity of pHT43-TPD construction was confirmed by restriction analysis and sequencing. Extracted pHT43-TPD was used for transformation of *B. subtilis* (WB600) by Eppendorf Eporator (Eppendorf, USA) according to the *B. subtilis* electro-transformation method 10. In order to ensure the accuracy of transformation, *B. subtilis* colonies were cultured on LB agar medium containing 5 *μg/ml* chloramphenicol as a selective marker and were verified by colony PCR. Plasmid DNA preparation, restriction enzyme digestion and agarose gel electrophoresis processes were performed in accordance with the published methods 11.

### Gene expression in Bacillus subtilis (WB600)

*B. subtilis* (WB600) cells, harbouring the recombinant vector pHT43 were grown on a LB-agar plate containing 5 *μg/ml* chlromphenicol at 37°*C*. A single colony was selected from the plate and cultured for 4 *hr* in 5 *ml* LB broth containing 5 *μg/ml* chloramphenicol at 37°*C* and 200 *rpm*. One millilitre of this culture was used to inoculate 15 *ml* of the same medium which was incubated under similar conditions. 0.1 *mM* of Isopropyl-D-thio-galactoside (IPTG) was added to induce expression when the Optical Density (OD) of the culture medium at a wavelength of 600 *nm* reached about 0.7.

Culture samples were withdrawn at different time intervals at 1 *ml*/*hr*. Cell pellet of samples was then sedimented by centrifugation at 10000 *rpm* for 10 *min*. Protein preparation process was carried out for each section separately. The procedure of protein precipitation from the supernatant liquid was performed by saturation with Polyethylene Glycol (PEG) 8000 followed by centrifugation at 10000 *rpm* for 20 *min* at 4°*C*. Subsequently, fidelity of the TPD protein samples prepared from the cultivation broth was checked by sodium dodecyl Sulphate-Poly-acrylamide gel (SDS-PAGE) and Western Blotting (WB). “ImageJ” software was used to estimate TPD expression level.

## Results

### Construction of pHT43-TPD

**Figure 3.**
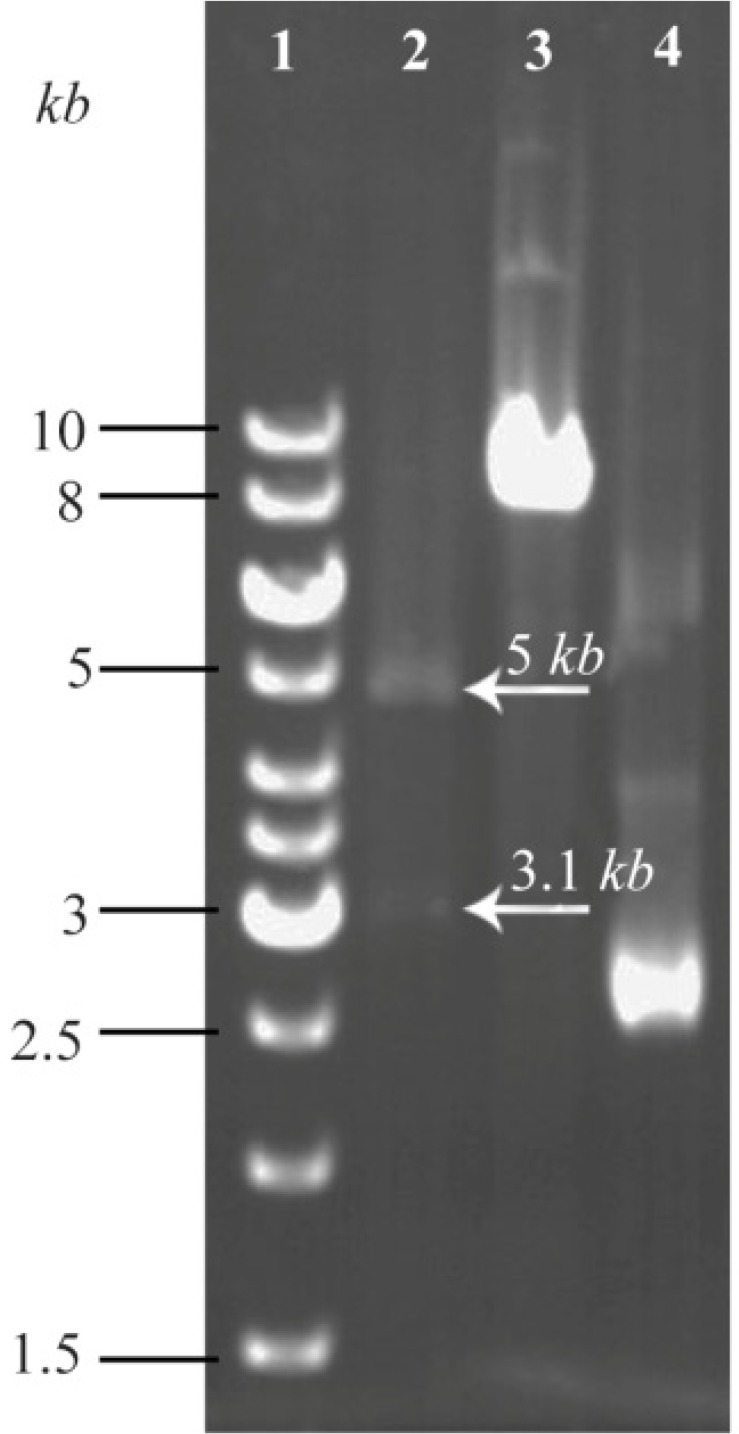
Restriction enzyme digestion confirming new recombinant of pHT43-TPD construct. Lane 1: GeneRuler™ 1 *kb* DNA ladder (Thermo, USA). Lane 2: Two bands of 5 *kb* and 3.1 *kb* generated from PstI/XhoI double digestion on the pHT43-TPD construct. As demonstrated in figure 1, a unique PstI site and a unique XhoI site are indentified through target DNA and plasmid backbone, respectively. Lane 3: A single band obtained from the same double digestion on non-recombinant plasmid (a negative control). Lane 4: undigested pHT43 plasmid.

Initially, agarose gel electrophoresis of the TPD PCR product indicated correct weight of 158 *bp* band. Analysis of sequencing results also confirmed the fidelity of TPD sequence fused with His-tag. Accuracy of pHT43 vector carrying TPD gene was rechecked by conducting colony PCR and enzymatic digestion analysis (Figure 3).

### Expression of TPD protein in Bacillus subtilis WB600

**Figure 4.**
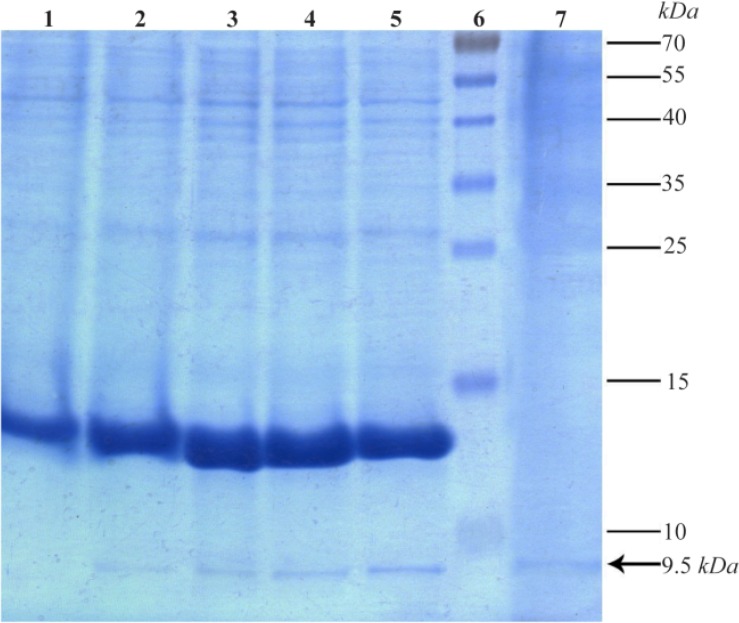
Expression and secretion analysis of the present recombinant TPD protein in cell pellet and culture medium samples by 15% acrylamide gel. Lane 1: Expression before induction. Lanes 2 to 5: Gradual increase of expression respectively from 1 to 4 *hr* after induction. Lane 6: Prestained Protein Ladder (Thermo, USA). Lane 7: Secretion of the recombinant protein in the medium 4 *hr* after induction. Position of the recombinant tag-fused TPD protein is shown with an arrow.

Results of SDS-PAGE for samples after induction of up to 4 *hr* indicated a gradual increase in intensity of 9.5 *kDa* band corresponding to target protein. Existence of the same band in a precipitated protein sample from 15 *ml* of the culture medium confirms the secretory expression of TPD protein (Figure 4). Relative expression level of TPD quantified by “ImageJ” software was at least 15.22% of total protein of *B. subtilis* WB600.

### Western blot analysis of TPD

**Figure 5.**
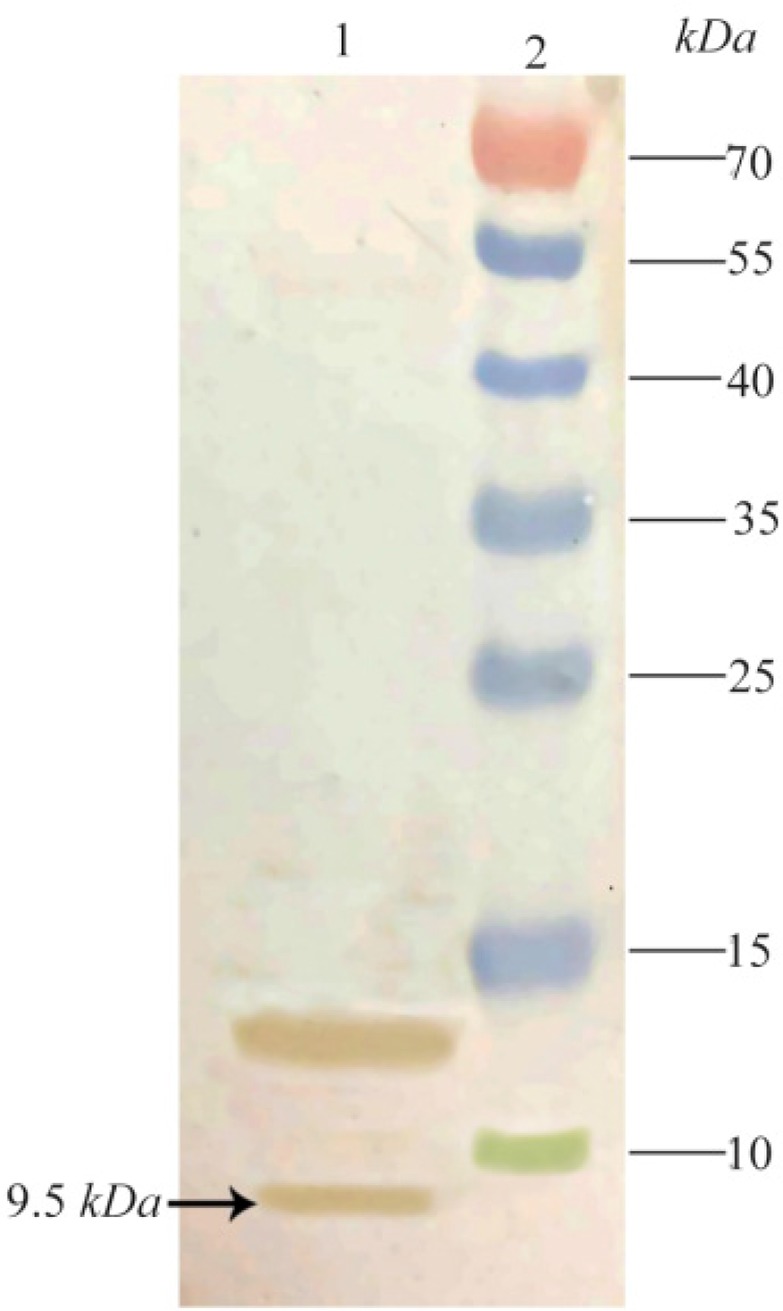
Outcome of performing a western blot to recheck the fidelity of TPD expression. Lane 1: Expression 4 *hr* after induction, Lane 2: Prestained Protein Ladder (Thermo, USA). The position of target band is shown with an arrow.

The fidelity of 9.5 *kDa* expressed TPD was reconfirmed with western blotting using nitrocellulose membrane and anti his-tag antibody as demonstrated in figure 5.

## Discussion

Osteoprosis as a bone disease is common. It could lead to patients expiration with certain severe fractures. Possibility of developing breast cancer is 1 in 9 among white women, while the incidence of hip fracture may be 1 in 6 during their lifetimes 12. Compared to rates recorded in 1990, the worldwide incidence of hip fracture is predicted to increase by 310 and 240% in men and women, respectively by 2050 13. Teriparatide as an osteoporosis treatment medication has been produced in both synthetic and recombinant forms. Several host systems such as *E. coli*, *Saccharomyces cerevisiae*, *Pichia pastoris*, and mammalian cell lines have been introduced to express this therapeutic polypeptide 14. Various approaches have been adopted to express human parathyroid hormone in *E. coli*, but this expression resulted in formation of large amounts of inactive proteins as inclusion bodies. Therefore, a prolonged protocol is required for their low-yield conversion into active proteins 15. Nevertheless, market available Forteo, that is produced in *E. coli* host 16 is a well known biopharmaceutical protein manufactured by Eli Lilly, France.

Existence of stable expression systems is a necessity for economical production of recombinant proteins. In comparison to *E. coli*, *B. subtilis* is a more attractive expression platform because it has a natural ability to interact with plants or pathogens via peptide secretion into the media 17. Furthermore, *B. subtilis* (WB600) is an appealing host for production of heterologous secretory proteins 9,18 due to absence of significant codon bias 19, deficiency of six extracellular proteases 20, lack of endotoxin (LPS), and existence of high secretory capacity for direct protein export into extracellular medium. This simplifies downstream purification and prevents the formation of inclusion bodies.

## Conclusion

Owing to above-mentioned advantages of *B. subtilis* over *E. coli* in production of recombinant proteins with pharmacological activities, feasibility of expression and secretion of TPD in *B. subtilis* have been examined in this study for the first time, even though the efficiency of expression was not high enough. It is accepted that every recombinant protein is unique and needs some levels of adaptation for production in different systems. Expression conditions must be optimized to enhance the yield of soluble protein. These optimizations must be carried out at both level of molecular regulations and fermentation conditions.

*B. subtilis* was taken as a candidate for TPD production through a new simplified and cost-effective approach in this research. Further studies are needed to optimize, purify and asses the biological activity of TPD expressed into *B. subtilis*.
